# Circular RNA: Biosynthesis *in vitro*

**DOI:** 10.3389/fbioe.2021.787881

**Published:** 2021-11-30

**Authors:** Xinjie Chen, Yuan Lu

**Affiliations:** Key Laboratory of Industrial Biocatalysis, Ministry of Education, Department of Chemical Engineering, Tsinghua University, Beijing, China

**Keywords:** circular RNA, RNA synthesis, *in vitro* transcription, enzymatic ligation, ligase, permuted intron-exon

## Abstract

Circular RNA (circRNA) is a unique type of noncoding RNA molecule. Compared with traditional linear RNA, circRNA is a covalently closed circle produced by a process called backsplicing. CircRNA is abundant in many cells and has rich functions in cells, such as acting as miRNA sponge, protein sponge, protein scaffold, and mRNA regulator. With the continuous development of circRNA study, circRNA has also played an important role in medical applications, including circRNA vaccines and gene therapy. In this review, we illustrate the synthesis of circRNAs *in vitro*. We focus on biological ligation methods, such as enzymatic ligation from the bacteriophage T4 and ribozyme method. In addition, we summarize the current challenges in the design, synthesis, application, and production of circRNAs, and propose possible solutions in the future. CircRNA is expected to play an essential role in basic research and medical applications.

## Introduction

A newly described RNA, called circular RNA (circRNA) (Sanger et al., 1976; Arnberg et al., 1980; Kos et al., 1986; Flores et al., 2011; Salzman et al., 2012; Jeck et al., 2013; Memczak et al., 2013; Guo et al., 2014; Jeck and Sharpless, 2014; Wang et al., 2014), has got much attention because of the development of high-throughput RNA-sequencing technology in recent years. Compared with traditional linear RNA, circRNA is a 3′-5′ covalently closed ring (Holdt et al., 2018) and does not need 5′-cap or 3′-poly(A) tails to keep it stable (Petkovic and Müller, 2015). These circRNAs have been found in a wide range of cells (Sanger et al., 1976; Arnberg et al., 1980; Kos et al., 1986; Salzman et al., 2012; Memczak et al., 2013; Wang et al., 2014), and most of them are noncoding RNAs (ncRNAs) (Santer et al., 2019). According to the components of circRNAs, circRNAs are classified as exonic (ecircRNA), exon-intron (EIcircRNA), or intronic (ciRNA) (He et al., 2021; Tao et al., 2021). Among them, ecircRNAs are the major circRNAs and are mainly produced by a process called backsplicing *in vivo* (Schindewolf et al., 1996; Salzman et al., 2012; Jeck et al., 2013; Memczak et al., 2013; Guo et al., 2014; Barrett et al., 2015; Starke et al., 2015; Szabo et al., 2015). EcircRNAs are mainly located in the cytoplasm and have various functions. The most well-known function of circRNAs is the microRNA (miRNA) sponge (Li et al., 2020; He et al., 2021), such as circRNA *CDR1as* and *Sry* (Hansen et al., 2013a; Memczak et al., 2013). Besides, circRNAs are also proved to serve as protein sponge (Ashwal-Fluss et al., 2014) and scaffold for protein complexes (Du et al., 2017a; Du et al., 2020). In addition, circRNAs also play a role in regulating the translation and stability of mRNA levels (Chen et al., 2020b; Huang et al., 2020) and the activity of proteins (Jeck et al., 2013; Ashwal-Fluss et al., 2014; Abdelmohsen et al., 2017; Du et al., 2017b). As biomarkers, circRNAs are also proved to be relative to the age-dependent neural accumulation in *drosophila* (Westholm et al., 2014). Moreover, circRNAs can also be involved in cancer or other diseases (Hansen et al., 2013b; Li et al., 2015; Qu et al., 2015).

With the development of RNA vaccines, the improvement of the stability of RNAs has been a great challenge. Fortunately, circRNAs show great potential ahead of this challenge. The structural advantage of circRNAs brings it higher stability, especially against the degradation of exonucleases (Wesselhoeft et al., 2018). Endogenously produced circRNAs are 2–5 times more stable than linear RNAs (Enuka et al., 2016; Holdt et al., 2018). Therefore, the possibility that circRNAs serve as vaccines has been the main concern for researchers. Although circRNAs lack the essential elements for cap-dependent translation, the discovery of internal ribosome entry sites (IRES) makes it possible for circRNAs to act as translation templates (Legnini et al., 2017; Pamudurti et al., 2017; Wesselhoeft et al., 2018). N^6^-methyladenosine (m^6^A) is also proved to promote the extensive translation of circRNAs (Yang et al., 2017). Based on these studies, circRNA vaccines are likely to become the dominant form of the next generation of vaccines.

In this review, we introduced the synthesis of circRNAs *in vitro* and focused on the challenges and opportunities of circRNA biosynthesis. Since circRNAs have unique structures and special functions different from linear RNAs, further study of circRNAs will help understand biogenesis and expand the application of circRNAs in biological therapy and vaccine research.

## A Brief History of circRNAs

**FIGURE 1:**
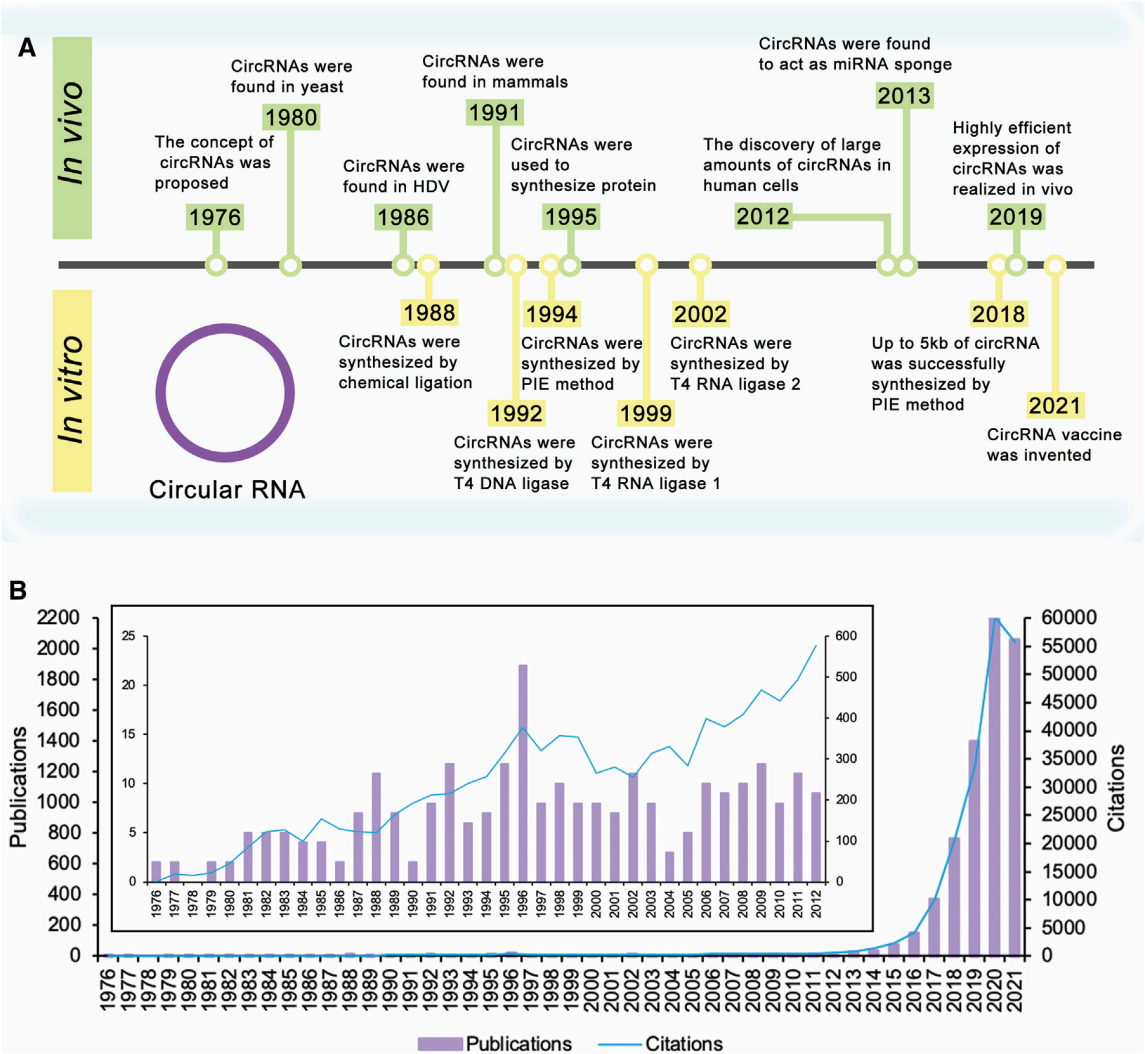
Timeline of circular RNA. **(A)** The concept of circRNAs was proposed in 1976. Extensive research on circRNAs began in 2012 with the discovery of large amounts of circRNAs in human cells. These researches have improved yields of circRNAs, realized the synthesis of large circRNA molecules, and expanded the applications of circRNAs. **(B)** Times cited and publications over time. There were a few publications about circRNAs before 2012. Since the discovery of large amounts of circRNAs in human cells in 2012, the number of publications and citations has increased year by year and is still rising.

The concept of circRNAs was first proposed by Sanger et al., in 1976 (Sanger et al., 1976) (Figure 1A). They studied four different highly purified viroids and found that viroids were covalently closed circRNA molecules. For the next 20 years, circRNAs were found in several kinds of life, such as yeast (Arnberg et al., 1980) and hepatitis delta virus (HDV) (Kos et al., 1986). CircRNAs were first discovered in mammals in 1991. Nigro et al. identified several abnormally spliced transcripts, in which exons from a candidate tumor suppressor gene (DCC) were scrambled during the splicing process *in vivo* (Nigro et al., 1991). After that, more circRNAs were found in mammalian cells (Cocquerelle et al., 1992; Capel et al., 1993; Zaphiropoulos, 1996; Zaphiropoulos, 1997; Surono et al., 1999). However, since the expression level of these circRNAs observed was so low, researchers believed that these circRNAs were the products of aberrant RNA splicing (Nigro et al., 1991; Cocquerelle et al., 1993). Therefore, researchers only knew the existence of circRNAs, but had no further understanding of theirs functions or impacts, and circRNAs did not receive much attention.

**FIGURE 2:**
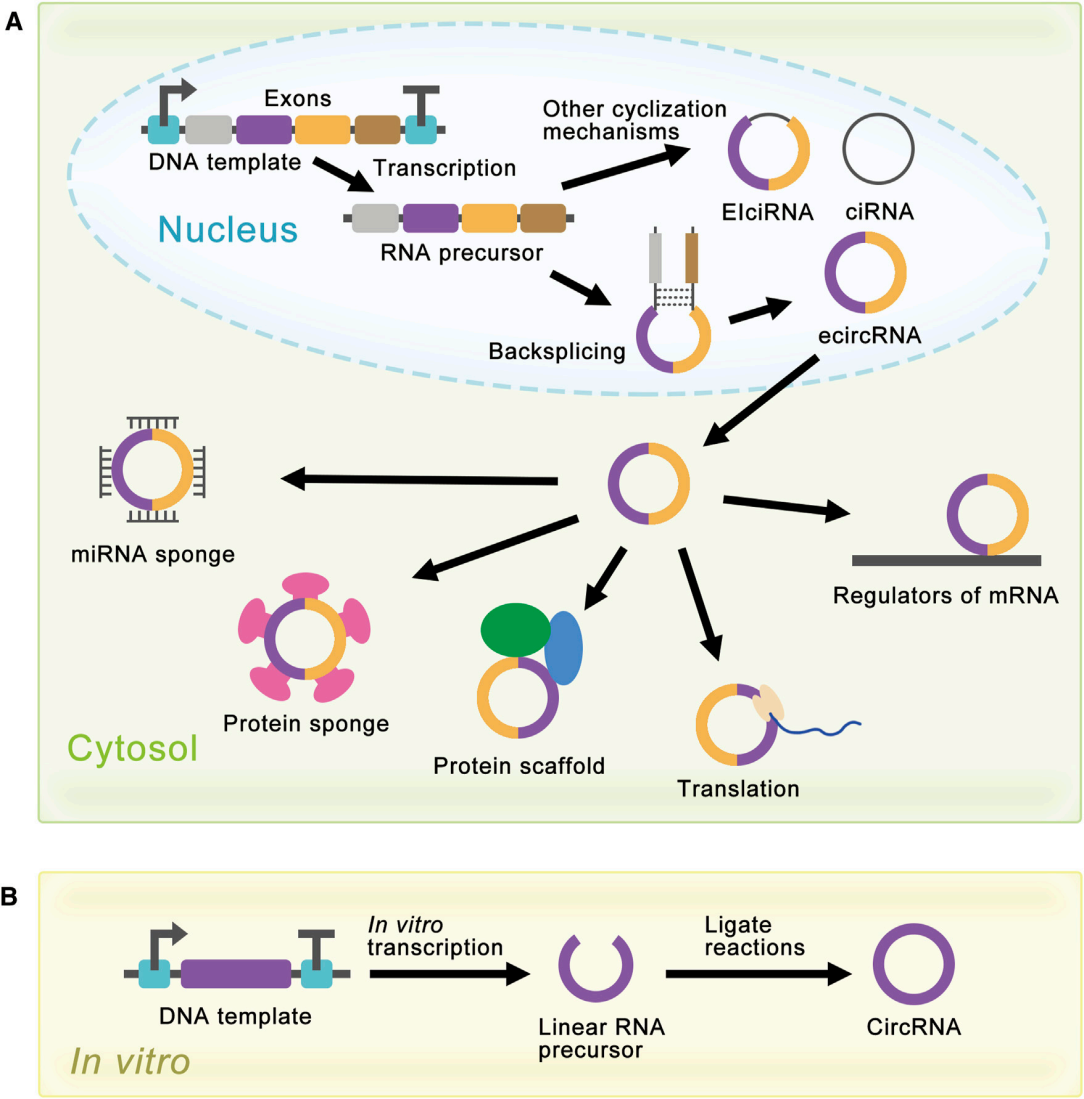
Schematic diagram of circRNA synthesis *in vivo* and *in vitro*. **(A)** EcircRNAs are the major circRNAs and are mainly produced by a process called backsplicing *in vivo*. EIciRNAs and ciRNAs are produced by other cyclization reactions and located in the nucleus. EcircRNAs are mainly located in the cytoplasm and have various functions. In the cytosol, circRNAs can act as miRNA sponges, protein sponges, protein scaffolds, translation templates, and regulators of mRNA. **(B)** Linear RNA precursor is produced from *in vitro* transcription, and researchers have developed several methods for linear RNA precursor ligation to synthesize circRNA *in vitro*.

Extensive research on circRNAs began in 2012 with the discovery of large amounts of circRNAs in human cells (Figure 1B). With the development of high-throughput sequencing technology and computational analysis, Salzman et al. found that a substantial fraction of the spliced transcripts from hundreds of genes were circRNAs by deep sequencing of RNA from a variety of normal and malignant human cells (Salzman et al., 2012). This result proved that the circRNA was not a mistake of aberrant RNA splicing, but a general feature of the gene expression program in human cells. The discovery revived great interest in circRNA research, and relative research was growing exponentially. In 2013, the function of circRNAs as the miRNA sponge was reported (Hansen et al., 2013a; Memczak et al., 2013). Besides, circRNAs were proved to be more stable than associated linear mRNAs *in vivo* (Jeck et al., 2013). The confirmation of the abundance, function, and stability of circRNAs laid the foundation of circRNAs research. Since then, more functions of circRNAs have been explored by researchers (Figure 2A).

Although natural circRNAs were ncRNAs and were considered incapable of translation, research had proved that manufactured circRNAs with IRES could be translated *in vivo* and *in vitro* (Chen and Sarnow, 1995; Perriman and Ares, 1998; Wang and Wang, 2015; Barrett and Salzman, 2016; Szabo and Salzman, 2016), which opened the way for the synthesis of proteins with circRNAs. More and more research was devoted to artificially controlling the expression level of circRNAs to realize the enrichment of circRNAs functions (Liang and Wilusz, 2014; Zhang et al., 2014; Kramer et al., 2015; Zhang et al., 2016; Garikipati et al., 2019). For the past few years, researchers have achieved highly efficient expression of circRNAs in cells using autocatalytic transcripts (Litke and Jaffrey, 2019) or viroid scaffolds (Daròs, 2021). At the same time, researchers had also developed several methods for the synthesis of circRNAs *in vitro*, such as chemical method (Sokolova et al., 1988), enzymatic method (Moore, 1999), and ribozyme method (Puttaraju and Been, 1992). These methods produced circRNAs by ligating the ends of linear RNA precursor, which would be covered later (Figure 2B).

## Synthesis of circRNAs *in vitro*

### Synthesis of Linear RNA Precursor *in vitro*

At present, the main method of circRNA synthesis *in vitro* is ligating the ends of linear RNA precursor to produce a covalently closed circle. Linear RNA can be produced by chemical synthesis (Müller and Appel, 2017; Obi and Chen, 2021) or enzymatic strategy (Obi and Chen, 2021). The advantage of chemical synthesis is that the 5′ monophosphate can be directly introduced during the synthesis process for future cyclization. However, limited by the high cost of purification and low yield, chemical synthesis can only produce RNA of less than 50 to 70 nucleotides in length. Therefore, enzymatic strategy is the primary linear RNA synthesis method at present. Enzymatic strategy is usually realized through an *in vitro* transcription (IVT) reaction (Beckert and Masquida, 2011), which includes DNA template, reaction buffer, and phage RNA polymerase. The phage RNA polymerase usually derives from the T7, SP6, or T3 bacteriophages, and T7 RNA polymerase (Rio, 2013) is the most common phage RNA polymerase. IVT reaction allows for longer RNA synthesis at a lower cost. However, the run-off nature of phage polymerases may result in incomplete RNA. Some studies have improved transcription quality and reduced side reactions by mutating wild-type phage RNA polymerase.

### Chemosynthesis or Biosynthesis?

Researchers have developed several methods for linear RNA precursor ligation *in vitro*. These ligation methods include chemical ligation, enzymatic ligation, and ribozyme method. Chemical ligation is realized by using cyanogen bromide (BrCN) or 1-ethyl-3-(3′-dimethylaminopropyl) carbodiimide to link DNA-RNA hybrids (Sokolova et al., 1988). However, this method suffers from low ligating efficiency (Dolinnaya et al., 1991) and biosafety concerns. In addition, chemical ligation forms 2′, 5′-phosphodiester bonds instead of the natural 3′, 5′-phosphodiester bonds (Table 1). Therefore, chemical ligation is not a common ligation method. Researchers are more interested in the biosynthesis of circRNAs, which includes enzymatic ligation and ribozyme method.

**TABLE 1:** Advantages and disadvantages of different ligation method.

Ligation method		Advantages	Disadvantages
Chemical ligation		• Only chemical reagents	• Low ligating efficiency
		• No biological components	• Biosafety concern
			• 2′, 5′-phosphodiester bonds
Enzymatic ligation	T4 DNA ligase	• Accurate	• Affected by significant RNA secondary structure
			• Affected by high percentage of Us
			• Low efficiency
			• Intermolecular end joining side reactions
	T4 RNA ligase 1	• High efficiency	• Low ligating efficiency for large RNA molecules
		• Synthesize as little as 6 to 8 nucleotides of circRNAs	• Affected by significant RNA secondary structure
			• Intermolecular end joining side reactions
	T4 RNA ligase 2	• More efficient for linear RNA precursor folding into a secondary structure with the ligation junction in a double-stranded region	• Low ligating efficiency for large RNA molecules
			• Intermolecular end joining side reactions
Ribozyme method	Group I intron self-splicing system	• Simple reaction condition and purification method	• Affected by significant RNA secondary structure
		• Can be used for RNA cyclization *in vitro* and *in vivo*	
		• Can synthesize large circRNAs	
	Group II intron self-splicing system	• Accurate ligation	• 2′, 5′-phosphodiester bonds
			• The mechanism remains unclear *in vitro*
	Hairpin ribozyme method	• High efficiency for small circRNAs	• Unstable
			• Exogenous HPR sequences

### Enzymes From Bacteriophage T4

Enzymatic ligations are realized by catalytic reactions of several enzymes from the bacteriophage T4, including T4 DNA ligase (T4 Dnl), T4 RNA ligase 1 (T4 Rnl 1), and T4 RNA ligase 2 (T4 Rnl 2). It is worth noting that linear RNA precursor needs a 3′-OH on the acceptor substrate and a 5′ monophosphate on the donor substrate for enzymatic ligation (Moore, 1999). If the linear RNA precursor is produced by chemical synthesis, a 5′ monophosphate can be incorporated during the synthesis or added after the synthesis using ATP and T4 polynucleotide kinase. However, if the linear RNA precursor is synthesized by IVT reaction, it usually starts with 5′-pppG. Therefore, the 5′-terminus has to be dephosphorylated prior using calf intestinal (CIP) enzyme or other phosphatases (Petkovic and Müller, 2018). Then, 5′ monophosphate can be added using ATP and T4 polynucleotide kinase. In addition, the addition of GMP to the IVT reaction mixture is also proved to be useful for phosphorylating the transcript at its 5′ end (Abe et al., 2018). However, this method must ensure that the first base in the transcript is G, so there are some limitations to this method.

**FIGURE 3:**
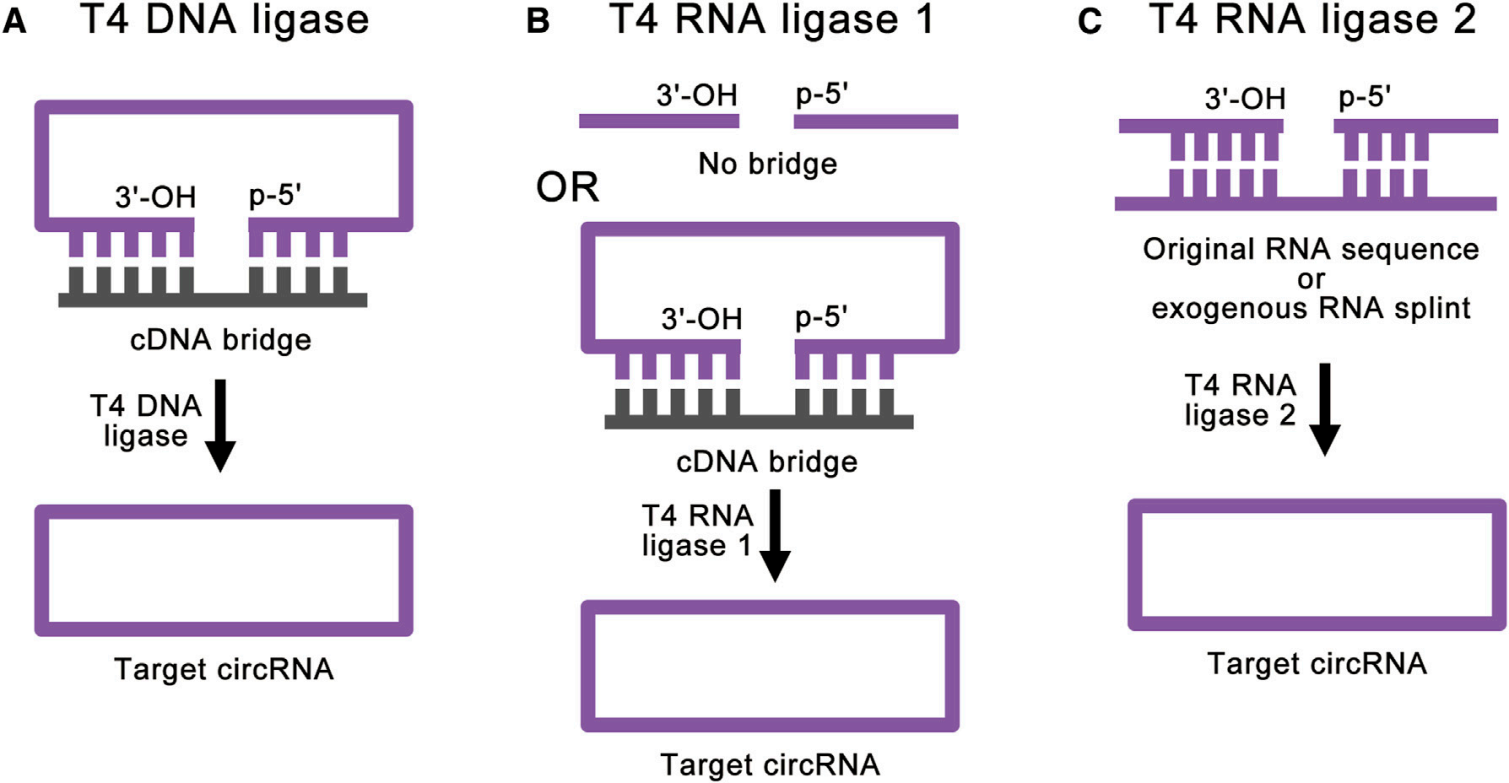
Strategies for enzymatic ligations. **(A)** T4 DNA ligase can help ligate double-stranded duplexes, such as DNA/RNA hybrids. With the help of cDNA bridge, T4 DNA ligase can achieve accurate RNA ligation. **(B)** T4 RNA ligase 1 catalyzes the nucleophilic attack of the 3′-OH terminus onto the activated 5′-terminus to form a covalent 5′, 3′-phosphodiester bond. The cDNA bridge can prevent the linear RNA precursor from folding into an unsuitable structure. **(C)** T4 RNA ligase 2 is suitable for linear RNA precursor with the ligation junction in a double-stranded region. With the help of RNA splint, T4 RNA ligase 2 can also realize the ligation of ends of ssRNA.

T4 Dnl ligation reaction includes inactivated kinase reaction and hybridized reaction. T4 Dnl can help ligate double-stranded duplexes, such as DNA/RNA hybrids (Moore, 1999) (Figure 3A). Therefore, this method needs a complementary DNA (cDNA) template or bridge to achieve RNA ligation. Generally, the cDNA bridge needs at least 10 nucleotides on either side of the ligation junction to guarantee high-quality ligation. The advantage of this method is that the accuracy of the linkage sites is greatly improved due to the addition of cDNA bridge. However, the ends of linear RNA precursor should be free of significant RNA secondary structure, and there should not be a high percentage of Us in the duplex region. Besides, T4 Dnl is less efficient in DNA/RNA hybrids linkage than double-stranded DNA (dsDNA) linkage. Due to these characteristics, only a few studies use this method for RNA ligation (Chen et al., 2017).

T4 Rnl 1 is a more common ligase for RNA ligation. T4 Rnl 1 catalyzes the nucleophilic attack of the 3′-OH terminus onto the activated 5′-terminus to form a covalent 5′, 3′-phosphodiester bond (Petkovic and Müller, 2018) and produces circRNAs (Figure 3B). Some studies have used cDNA bridge to prevent the linear RNA precursor from folding into an unsuitable structure (Wang and Ruffner, 1998). It is worth noting that T4 Rnl 1 has different preferences for the nucleotides of the 5′-terminus and 3′-terminus: A > G ≥ C > U for the 3′-terminal nucleotide acceptor, and pC > pU > pA > pG for the 5′-terminal nucleotide donor (England and Uhlenbeck, 1978; McLaughlin et al., 1982; Petkovic and Müller, 2015; Müller and Appel, 2017). In this way, as little as 6 to 8 nucleotides of circRNAs can be synthesized (Kaufmann et al., 1974; Petkovic and Müller, 2015). This method can achieve high-efficiency single-stranded RNA (ssRNA) linkage. However, the RNA ligation efficiency reduces with large RNA molecules (Costello et al., 2020). Similar to the T4 Dnl ligation reaction, significant RNA secondary structure at the ends of linear RNA precursor can greatly reduce the ligation efficiency of T4 Rnl 1. Besides, intermolecular end joining (oligomerization) is also a serious side reaction, which cannot be completely suppressed (Petkovic and Müller, 2018). This side reaction will increase with the increase of the concentration of linear RNA precursor, which limits the amount of RNA cyclization.

T4 Rnl 2 can also be used for RNA ligation (Ho and Shuman, 2002; Nandakumar and Shuman, 2004; Yin et al., 2004). Similar to T4 Rnl 1, T4 Rnl 2 also catalyzes the nucleophilic attack of the 3′-OH terminus onto the activated 5′-terminus to form a covalent 5′, 3′-phosphodiester bond. However, T4 Rnl 2 is much more active at joining nicks in double-stranded RNA (dsRNA) than at ligating the ends of ssRNA (Nandakumar et al., 2004; Bullard and Bowater, 2006) (Figure 3C). Based on this feature, when the linear RNA precursor folds into a secondary structure with the ligation junction in a double-stranded region, the efficiency of T4 Rnl 2 is much higher than that of T4 Rnl 1 (Petkovic and Müller, 2018). Besides, with the help of RNA splint, T4 Rnl 2 can also realize the ligation of ends of ssRNA. However, RNA splints cannot be used to ligate short RNA precursors, because the splint-precursor complex is sterically unstable when the length of linear RNA precursor is shorter than 30 nucleotides. Same as T4 Rnl 1, T4 Rnl 2 also suffers from low efficiency for large RNA molecules and side reactions. To overcome these challenges, there are studies using software to simulate the secondary structure of the target circRNA and hypothetically cut at one site so that a few intramolecular base pairs are formed at the terminal (Chen et al., 2020a). This method can achieve efficient RNA cyclization at high concentrations with T4 Rnl 2, but it is still affected by different RNA sequences.

In a word, T4 Dnl and T4 Rnl 1 are suitable for RNA ligations without complex secondary structures. T4 Rnl 2 is more suitable for linear RNA precursor with the ligation junction in a double-stranded region. Therefore, different T4 ligases need to be selected according to the secondary structure of the linear RNA precursor. However, all these enzymatic ligation methods cannot realize large RNA molecules ligation, and cannot totally avoid intermolecular end-joining side reactions. These problems still need to be solved (Table 1).

### Ribozyme Method

**FIGURE 4:**
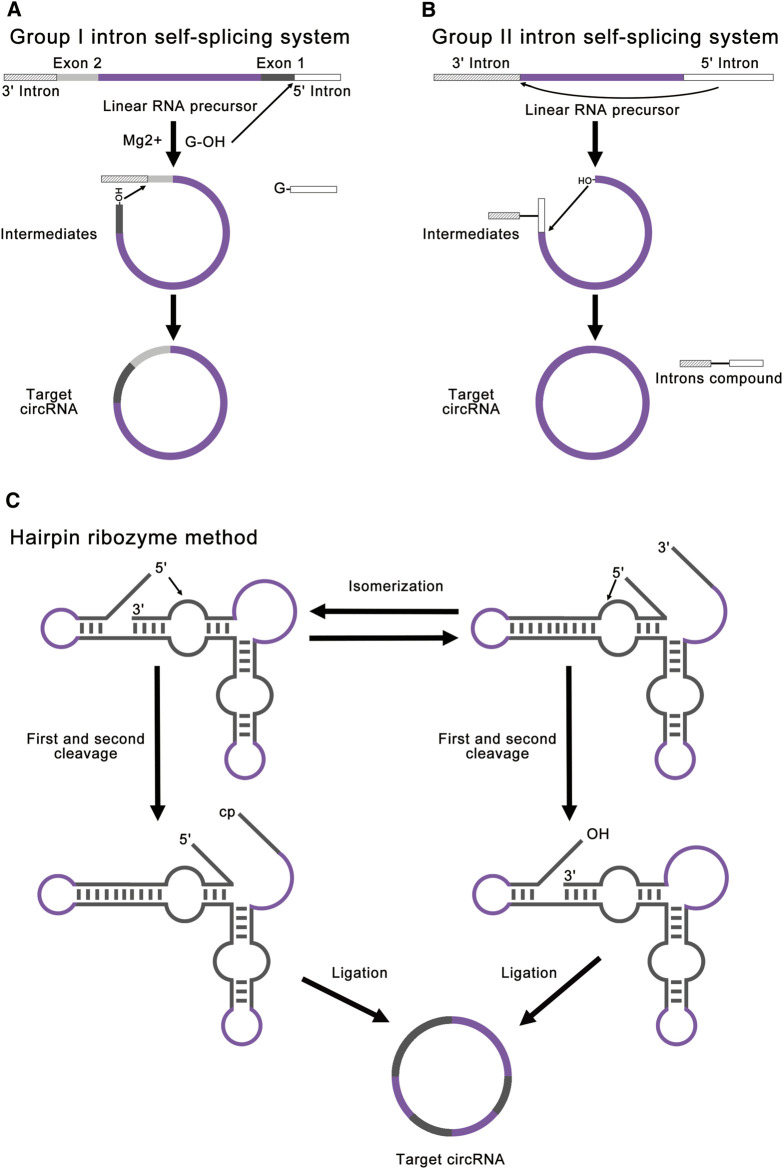
Strategies for ribozyme methods. **(A)** Group I intron self-splicing system requires only the addition of GTP and Mg^2+^ as cofactors and shows great potential for protein synthesis. This method realized RNA ligation through a normal group I intron self-splicing reaction, including two transesterifications at defined splice sites. The final circRNA will contain exogenous exon sequences. **(B)** Group II intron self-splicing system involves the joining of the 5′ splice site at the end of an exon to the 3′ splice site at the beginning of the same exon. All exon sequences are dispensable for group II intron catalyzed inverse splicing. This method can enable more accurate linear RNA precursor ligation. **(C)** Hairpin ribozyme method can produce circRNA through the rolling circle reaction and the self-splicing reaction. The linear RNA precursor with HPR will fold into two alternative cleavage-active conformations to remove the 3′-end and the 5′-end. As a result, the intermediate will contain a 5′-OH and a 2′, 3′-cyclic phosphate to produce target circRNA.

Modified group I intron self-splicing system is the most common ribozyme method, which is also called permuted introns and exons (PIE) method (Puttaraju and Been, 1992; Ford and Ares, 1994; Umekage and Kikuchi, 2009; Wesselhoeft et al., 2018; Rausch et al., 2021). PIE method requires only the addition of GTP and Mg^2+^ as cofactors and shows great potential for protein synthesis. This method realized RNA ligation through a regular group I intron self-splicing reaction, including two transesterifications at defined splice sites (Figure 4A). PIE method can be used for RNA cyclization *in vitro* and *in vivo* (Meganck et al., 2021), which broadens its application. Compared to chemical ligation and enzymatic ligation, PIE method could be applied for the cyclization of larger linear RNA precursor, and the reaction condition and purification method of PIE method is simpler. A recent study has realized accurate RNA ligation by designing custom-tailored PIE transcription templates from which synthetic circRNAs of almost any sequence may be efficiently synthesized without exogenous exon sequences (Rausch et al., 2021). Based on these advantages, PIE method is currently the most studied and most widely used RNA ligation method. However, PIE method still has its disadvantages. Due to the complexity of RNA secondary structure, different RNA sequences will lead to a great difference in the final circRNA field, which limits the application of PIE method. Due to the introduction of exogenous exon sequences, the final circRNA sequence will be different from the original linear RNA precursor sequence, which may negatively affect the validation of some circRNA functions.

Group II introns can also be used for circRNA synthesis, which involves an inverse splicing reaction (Jarrell, 1993; Mikheeva et al., 1997). This splicing reaction involves the joining of the 5′ splice site at the end of an exon to the 3′ splice site at the beginning of the same exon (Figure 4B). Compared to the group I introns, all exon sequences are dispensable for group II intron-catalyzed inverse splicing. Therefore, this method can enable more accurate linear RNA precursor ligation. However, this method forms 2′, 5′-phosphodiester bonds at the ligation site instead of the natural 3′, 5′-phosphodiester bonds, and the mechanism remains unclear *in vitro* (Petkovic and Müller, 2015; Müller and Appel, 2017; Obi and Chen, 2021). At present, there are few studies concentrating on group II introns.

Hairpin ribozyme (HPR) can produce circRNAs through rolling circle reaction and the self-splicing reaction from circular single-strand DNA template (Diegelman and Kool, 1998; Kazakov et al., 2006; Dallas et al., 2008; Petkovic and Müller, 2013). The linear RNA precursor with HPR will fold into two alternative cleavage-active conformations to remove the 3′-end and the 5′-end. As a result, the intermediate will contain a 5′-OH and a 2′, 3′-cyclic phosphate to produce the target circRNA (Figure 4C). In this method, small circRNAs can be produced from long repeating RNAs transcribed by RNA polymerase through a rolling circle mechanism *in vitro* (Diegelman and Kool, 1998). This method is mainly used for efficient production of small circRNAs (Hieronymus and Müller, 2019). Freezing stimulation, ionic conditions, and additional cofactors are proved to affect the activity of HPR (Nesbitt et al., 1999; Kazakov et al., 2006; Strohbach et al., 2006; Dallas et al., 2008). The disadvantage of this method is that the circRNA is not stable due to the dynamic equilibrium of HPR-catalyzed cleavage and ligation (Müller and Appel, 2017). Besides, the circRNA contains HPR sequences, which may negatively affect the function of some circRNAs.

In a word, PIE method using group I introns is suitable for most circRNAs production at present, which is still limited by the secondary structure of linear RNA precursor. Group II introns method can enable more accurate linear RNA precursor ligation, but the mechanism remains unclear *in vitro*. HPR method can efficiently produce small circRNAs but suffers from unstable product and exogenous HPR sequences. Among these ribozyme methods, PIE method shows the greatest potential for circRNAs production. At present, PIE method has been widely used in basic research and industrial production (Table 1).

## Challenges and Outlooks

Although there are many ways to synthesize circRNAs *in vitro*, there are still many challenges and opportunities. CircRNA is still a hot area of research, and there are many difficulties to be solved (Table 2).

**TABLE 2:** Challenges and potential solutions for circRNAs.

Challenges	Potential solutions
Secondary structure of linear RNA precursor	o Unnatural nucleotides
	o RNA-binding proteins
Cyclization efficiency, especially for large RNA molecules	o Mutating the wild enzyme from the bacteriophage T4
	o Rational design
Side reactions, especially for intermolecular end joining reaction	o Optimizing the cyclization reaction conditions and controlling the linear RNA precursor concentration
	o Immobilizing the ligase with hydrogels or other materials
Production of modified circRNAs	o Using unnatural nucleotides for the synthesis of linear RNA precursor
	o Incorporating chemically modified groups or unnatural nucleotides during the ligation reaction
Untapped potential	o Transferring scientific research achievements to commercial application
	o Further exploring the function and mechanism of circRNAs
The yield of circRNAs	o Optimizing current reaction components and conditions
	o New type reactors
The cost of raw materials	o Using cells to synthesize nucleotides or directly synthesize linear RNA precursors

Although PIE method has realized accurate linear RNA precursor ligation (Rausch et al., 2021), the structure of the linear RNA precursor still has a marked effect on circRNA synthesis yield. To overcome this problem, embedding unnatural nucleotides into linear RNA precursor may help change its secondary structure and improve the efficiency of ligation. Besides, RNA-binding proteins (RBPs) may also help the cyclization of RNA.

In terms of enzyme design, although existing natural enzymes from the bacteriophage T4 are capable of RNA cyclization, there is still much room for improvement. It is necessary to improve the cyclization efficiency of the ligases by mutation or rational design, especially for large RNA molecules. The critical point of mutation and rational design is to improve the binding efficiency of ligase to substrate RNA and the selectivity of cyclic products.

Reducing side reactions during circRNAs synthesis is also a big challenge. At present, the main side reaction is the intermolecular end-joining reaction, which exists in most circRNA synthesis methods and cannot be completely avoided. Therefore, it is necessary to optimize the cyclization reaction conditions and control the linear RNA precursor concentration to reduce the occurrence of such side reactions. In addition, immobilizing the ligase with hydrogels or other materials may realize the compartmentalization of the reaction and the cyclization of a single RNA substrate.

With the growing application of circRNAs, the synthesis of circRNAs with chemically modified or unnatural nucleotides is also difficult to be solved. Since circRNA does not have a terminal structure, the modification of circRNA is more difficult than linear RNA. One solution is to use unnatural nucleotides for the synthesis of linear RNA precursors, which can embed unnatural nucleotides into the final circRNA to improve the stability of circRNA and diversify its functions. In addition, the incorporation of chemically modified groups or unnatural nucleotides during the ligation reaction is also a possible solution.

Although circRNAs have been proved to hold the potential to become a novel vaccine (Qu et al., 2021), the commercial potential of circRNAs has yet to be fully exploited. Because of the unique structure of circRNAs, circRNAs can be used in the synthesis of some specific proteins, such as protein materials with lots of repeated sequences. Besides, circRNA can also be used as an expression template in cell-free expression systems. At the same time, the function of circRNA as ncRNA is also important. CircRNAs can not only be used as biomarkers of some diseases but also be used for gene therapy (Holdt et al., 2018). Since the function and mechanism of circRNAs in cells have not been fully studied, applications for circRNAs are still being developed. Further efforts are still needed.

Faced with the growing demand for circRNAs, large quantities of circRNAs need to be produced. Generally, increasing the yield of circRNAs requires increasing the concentration of the ligation reaction components, such as linear RNA precursors. However, an increase in the concentration of linear RNA precursors will lead to an increase in side reactions. These side reactions will decrease the yield of target circRNAs and increase the difficulty of product purification. Therefore, further optimization of current reaction components and conditions may be required based on scaled-up production systems. This problem may also be solved by new-type reactors, such as microreactors. At the same time, the cost of current circRNAs production is still a concern for researchers. The main reason is the cost of raw materials, such as expensive nucleotides. One potential solution is to use cells to synthesize nucleotides or directly synthesize linear RNA precursors. However, how to efficiently purify these raw materials produced by cells still needs further research.

In a word, circRNA is still a hot area of current research and holds great potentials. With the continuous efforts in this field, circRNAs will play an essential role in basic research and medical applications, and become an important part of human health in the near future.
